# List No. 4 of Training-Schools of Registered Nurses

**Published:** 1918-02-16

**Authors:** 


### THE COLLEGE OF NURSING

**(Limited by Guarantee).**

### List No. 4 of Training-Schools of Registered Nurses.

In recent issues we have published the names of the training-schools where nurses had trained who at the Council meetings of the College of Nursing have been passed as members of the College, holding certificates of the training-schools appearing on the list. It is hardly credible, but it appears that there is so little apprehension in the minds of some readers of the meaning of a paragraph, that letters have been received from institutions expressing disappointment that their name has not appeared on a particular list, when in fact no application for membership had been received at the particular Council meeting from any nurse holding the certificate of such institution. The misapprehension appears to have arisen because the inquirers have got it into their head that the list of training-schools represented by applicants for registration at a particular Council meeting includes the whole of the recognised nurse-training schools throughout the country; and therefore when the reader does not find on each list the name of her institution she immediately fears that it has ceased to be a recognised training-school. We hope, therefore, that all readers will apprehend this time that the following list contains the names of the training-schools to which applicants for membership were passed at the last Council meeting of the College of Nursing as the holders of a certificate from one of the undermentioned schools:—

Great Northern Central Hospital, N.; Guy's Hospital, S.E.; King's College Hospital, S.E.; London Hospital, E.; Metropolitan Hospital, London; Middlesex Hospital, W.; Miller Hospital, Greenwich, S.E.; Poplar Hospital, E.; Prince of Wales' General Hospital, N.; St. Bartholomew's Hospital, E.C.; St. George's Hospital, S.W.; St. Mary's Hospital, W.; St. Thomas's Hospital, S.E.; University College Hospital, W.C.; Bermondsey Infirmary, London; Bethnal Green Infirmary, London; City of London Infirmary; Croydon Infirmary; Greenwich Infirmary, Vanbrugh Hill; Hammersmith Infirmary, London; Brentford Infirmary, Isleworth, W.; Kensington Infirmary, St. Mary Abbots; St. Giles's Infirmary, Camberwell; St. Marylebone Infirmary, London; West Ham Infirmary, Leytonstone; General Hospital, Birmingham; Queen's Hospital, Birmingham; Bolton Infirmary and Dispensary, Lancs; Bradford Royal Infirmary; General Hospital, Bristol; Royal Infirmary, Bristol; General Infirmary, Burton-on-Trent; General Hospital, Cheltenham; Royal Infirmary, Chester; Essex County Hospital, Colchester; Derbyshire Royal Infirmary, Derby; Royal Albert Hospital, Devonport; Guest Hospital, Dudley; Royal Devon and Exeter Hospital, Exeter; Royal Infirmary, Gloucester; County Hospital, Huntingdon; Royal Infirmary, Leicester,; General Infirmary, Leeds; Victoria Central Hospital, Liscard; Royal Infirmary, Liverpool; Royal Southern Hospital, Liverpool; West Kent General Hospital, Maidstone; Royal Infirmary, Manchester; Royal Hospital, Salford; General Hospital, Northampton; Radcliffe Infirmary, Oxford; General Hospital, Nottingham; Royal Hospital, Portsmouth; General Infirmary, Salisbury; Royal Infirmary, Sheffield; Royal South Hants Hospital, Southampton; The Infirmary, Stockport; Royal Infirmary, Sunderland; Clayton Hospital, Wakefield, Yorks; Royal Albert Edward Infirmary, Wigan; County Hospital, York; General Infirmary, Stafford; Eastville Poor-Law Infirmary, Bristol; Crosland Moor Poor-Law Infirmary, Huddersfield; St. Luke's Poor-Law Infirmary, Halifax; North Evington Poor-Law Infirmary, Leicester; Brownlow Hill Poor-Law Infirmary, Liverpool; Mill Road Infirmary, Liverpool; Chorlton Poor-Law Infirmary; Crumpsall Poor-Law Infirmary, Manchester; Newcastle-on-Tyne Union Infirmary; Harton Poor-Law Infirmary, South Shields; Walsall Poor-Law Infirmary, Pleck Road; Dudley Road Infirmary, Birmingham; General Hospital, Hobart, Tasmania; Dunedin Hospital, New Zealand; Brisbane General Hospital, Queensland, Australia; Royal Waterloo Hospital, S.E., and Seamen's Hospital, Greenwich; Royal Infirmary, Edinburgh; Western Infirmary, Glasgow; Royal Infirmary, Glasgow; Victoria Infirmary, Glasgow; Royal Infirmary, Aberdeen; Royal Infirmary, Dundee; Royal Infirmary, Perth; Royal Alexandra Infirmary, Paisley; Dumfries and Galloway Royal Infirmary; County Hospital, Ayr; Kilmarnock Infirmary; Chalmers Hospital, Edinburgh; Eastern District Hospital, Glasgow; Stobhill Hospital, Glasgow; Merryflatts Hospital, Govan; Eastern Hospital, Dundee.

For "A Complete System of Nursing," Reviewed by a Physician and a Trained Nurse, see p. 428;
State Registration: a Remarkable Meeting at Liverpool, see p. 424;
Women as Sanatoria Superintendents, see p. iv.

